# *F*-Expansion Method and New Exact Solutions of the Schrödinger-KdV Equation

**DOI:** 10.1155/2014/534063

**Published:** 2014-01-29

**Authors:** Ali Filiz, Mehmet Ekici, Abdullah Sonmezoglu

**Affiliations:** ^1^Department of Mathematics, Faculty of Science and Arts, Adnan Menderes University, 09010 Aydin, Turkey; ^2^Department of Mathematics, Faculty of Science and Arts, Bozok University, 66100 Yozgat, Turkey

## Abstract

*F*-expansion method is proposed to seek exact solutions of nonlinear evolution equations. With the aid of symbolic computation, we choose the Schrödinger-KdV equation with a source to illustrate the validity and advantages of the proposed method. A number of Jacobi-elliptic function solutions are obtained including the Weierstrass-elliptic function solutions. When the modulus *m* of Jacobi-elliptic function approaches to 1 and 0, soliton-like solutions and trigonometric-function solutions are also obtained, respectively. The proposed method is a straightforward, short, promising, and powerful method for the nonlinear evolution equations in mathematical physics.

## 1. Introduction

Nonlinear evolution equations are widely used to describe complex phenomena in many scientific and engineering fields, such as fluid dynamics, plasma physics, hydrodynamics, solid state physics, optical fibers, and acoustics. Therefore, finding solutions of such nonlinear evolution equations is important. However, determining solutions of nonlinear evolution equations is a very difficult task and only in certain cases one can obtain exact solutions. Recently, many powerful methods to obtain exact solutions of nonlinear evolution equations have been proposed, such as the inverse scattering method [1], the Bäcklund transformation method [2, 3], the Hirota bilinear scheme [4, 5], the Painlev expansion [6], the homotopy perturbation method [7, 8], the homogenous balance method [9], the variational method [10–12], the tanh function method [13–16], the trial function and the sine-cosine method [17], (*G*′/*G*)-expansion method [18, 19], the trial equation method [20–28], the auxiliary equation method [29], the Jacobian-elliptic function method [30–33], the *F*-expansion method [34–38], and the Exp-function method [39–42].

In the present research, we shall apply the the *F*-expansion method to obtain 52 types of exact solution: six for the Weierstrass-elliptic function solutions and the rest for Jacobian-elliptic function solutions of the Schrdinger-KdV equation:

(1) $$\begin{array}{c} iu_{t}=u_{xx}+uv,\;\;v_{t}+6vv_{x}+v_{xxx}={\left({\left|u\right|}^{2}\right)}_{x}. \end{array}$$

Among the methods mentioned above, the auxiliary equation method [29] is based on the assumption that the travelling wave solutions are in the form

(2) $$\begin{array}{c} u\left(\eta \right)=\sum_{i=0}^{n}a_{i}z^{i}\left(\eta \right),\;\eta =\alpha \left(x-\beta t\right), \end{array}$$

where *z*(*η*) satisfies the following auxiliary ordinary differential equation:

(3) $$\begin{array}{c} {\left(\frac{dz}{d\eta }\right)}^{2}=az^{2}\left(\eta \right)+bz^{3}\left(\eta \right)+cz^{4}\left(\eta \right), \end{array}$$

where *a*, *b*, and *c* are real parameters. Although many exact solutions were obtained in [29] via the auxiliary equation (3), all these solutions are expressed only in terms of hyperbolic and trigonometric functions. In this paper, we want to generalize the work in [29]. We propose a new auxiliary equation which has more general exact solutions in terms of Jacobian-elliptic and the Weierstrass-elliptic functions. Moreover, many exact solutions in terms of hyperbolic and trigonometric functions can be also obtained when the modulus of Jacobian-elliptic functions tends to one and zero, respectively.

The rest of the paper is arranged as follows. In Section 2, we briefly describe the auxiliary equation method (*F*-expansion method) for nonlinear evolution equations. By using the method proposed in Section 2, Jacobian-elliptic and the Weierstrass-elliptic functions solutions are presented in Sections 3 and 4, respectively. Soliton-like solutions and trigonometric-function solutions are listed in Sections 5 and 6, respectively. Some conclusions are given in Section 7. The paper is ended by Appendices A–D which play an important role in obtaining the solutions.

## 2. Description of the *F*-Expansion Method

Consider a nonlinear partial differential equation (PDE) with independent variables *x*, *t* and dependent variable *u*:

(4) $$\begin{array}{c} N\left(u,u_{t},u_{x},u_{xx},\ldots \right)=0. \end{array}$$

Assume that *u*(*x*, *t*) = *u*(*ξ*), where the wave variable *ξ* = *x* + *ct*. By this, the nonlinear PDE (4) reduces to an ordinary differential equation (ODE):

(5) $$\begin{array}{c} N\left(u,cu^{\prime },u^{\prime },u^{\prime \prime },\ldots \right)=0. \end{array}$$

Then we seek its solutions in the form

(6) $$\begin{array}{c} u\left(\xi \right)=\sum_{i=0}^{m}a_{i}F^{i}\left(\xi \right), \end{array}$$

where *a*_*i*_, *i* = 0,1, 2,…, *m*, are constants to be determined, *m* is a positive integer which can be evaluated by balancing the highest order nonlinear term(s) and the highest order partial derivative of *u* in (4), and *F*(*ξ*) satisfies the following auxiliary equation:

(7) $$\begin{array}{c} F^{\prime }\left(\xi \right)=\sigma \sqrt{PF^{4}\left(\xi \right)+QF^{2}\left(\xi \right)+R}, \end{array}$$

where *σ* = ±1 and *P*, *Q*, and *R* are constants. The last equation hence holds for *F*(*ξ*):

(8) F′′=2PF3+QF,F′′′=(6PF2+Q)F′,F(4)=24P2F5+20PQF3+(12PR+Q2)F,F(5)=(120P2F4+60PQF2+12PR+Q2)F′⋮

In Appendices A and B, we present 52 types of exact solution for (7) (see [34–37, 43] for details). In fact, these exact solutions can be used to construct more exact solutions for (1).

## 3. New Exact Jacobian-Elliptic Function Solutions of the Schrödinger-KdV Equation

The coupled Schrödinger-KdV equation

(9) $$\begin{array}{c} iu_{t}-u_{xx}-uv=0,\;\;v_{t}+6vv_{x}+v_{xxx}-{\left({\left|u\right|}^{2}\right)}_{x}=0 \end{array}$$

is known to describe various processes in dusty plasma, such as Langmuir, dust-acoustic wave, and electromagnetic waves [44–47]. Exact solution of (9) was studied by many authors [48–51]. Here the *F*-expansion method is applied to system (9) and gives some new solutions. Let

(10) u=eiθU(ξ),  v=V(ξ),θ=αx+βt,  ξ=x+ct,

where *α*, *β*, and *c* are constants.

Substituting (10) into (9), we find that *c* = 2*α* and *V*, *U* satisfy the following coupled nonlinear ordinary differential system:

(11) U′′+(β−α2)U+UV=0,2αV′+6VV′+V′′′−(U2)′=0.

Balancing the highest nonlinear terms and the highest order derivative terms in (11), we find *m* = 2 and *n* = 2. Therefore, we suppose that the solution of (11) can be expressed by

(12) $$\begin{array}{c} U\left(\xi \right)=a_{0}+a_{1}F\left(\xi \right)+a_{2}F^{2}\left(\xi \right),\\ V\left(\xi \right)=b_{0}+b_{1}F\left(\xi \right)+b_{2}F^{2}\left(\xi \right), \end{array}$$

where *a*_0_, *a*_1_, *a*_2_, *b*_0_, *b*_1_, and *b*_2_ are constants to be determined later and *F*(*ξ*) is a solution of ODE (7). Inserting (12) into (11) with the aid of (7), the left-hand side of (11) becomes polynomials in *F*(*ξ*) if canceling *F*′ and setting the coefficients of the polynomial to zero yields a set of algebraic equations, *a*_0_, *a*_1_, *a*_2_, *b*_0_, *b*_1_, and *b*_2_. Solving the system of algebraic equations with the aid of Mathematica, we obtain

(13) a0=0,  a1=±2P(Q−α−3α2+3β),  a2=0,b0=α2−β−Q,  b1=0,  b2=−2P.

Substituting these results into (12), we have the following formal solution of (11):

(14) $$\begin{array}{c} U=\pm 2\sqrt{P\left(Q-\alpha -3\alpha^{2}+3\beta \right)}F\left(\xi \right),\\ V=\alpha^{2}-\beta -Q-2PF^{2}\left(\xi \right),\;\text{where}\;\xi =x+ct. \end{array}$$

With the aid of Appendix A and from the formal solution of (14) along with (10), one can deduce more general combined Jacobian-elliptic function solutions of (1). Hence, the following exact solutions are obtained.

**Case 1**

*P* = *m* ^2^, *Q* = −(1 + *m* ^2^), *R* = 1, *F*(*ξ*) = *snξ*,

(15) u1=eiθ{±2m−1−m2−α−3α2+3βsnξ},v1=α2−β+1+m2−2m2sn2ξ.

**Case 2**

*P* = *m* ^2^, *Q* = −(1 + *m* ^2^), *R* = 1, *F*(*ξ*) = *cdξ*,

(16) u2=eiθ{±2m−1−m2−α−3α2+3βcdξ},v2=α2−β+1+m2−2m2cd2ξ.

**Case 3**

*P* = −*m* ^2^, *Q* = 2*m* ^2^ − 1, *R* = 1 − *m* ^2^, *F*(*ξ*) = *cnξ*,

(17) u3=eiθ{±2m−2m2+1+α+3α2−3βcnξ},v3=α2−β−2m2+1+2m2cn2ξ.

**Case 4**

*P* = −1, *Q* = 2 − *m* ^2^, *R* = *m* ^2^ − 1, *F*(*ξ*) = *dnξ*,

(18) $$\begin{array}{c} u_{4}=e^{i\theta }\left\{\pm 2\sqrt{-2+m^{2}+\alpha +3\alpha^{2}-3\beta }dn\xi \right\},\\ v_{4}=\alpha^{2}-\beta -2+m^{2}+2dn^{2}\xi . \end{array}$$

**Case 5**

*P* = 1, *Q* = −(1 + *m* ^2^), *R* = *m* ^2^, *F*(*ξ*) = *nsξ*,

(19) $$\begin{array}{c} u_{5}=e^{i\theta }\left\{\pm 2\sqrt{-1-m^{2}-\alpha -3\alpha^{2}+3\beta }ns\xi \right\},\\ v_{5}=\alpha^{2}-\beta +1+m^{2}-2ns^{2}\xi . \end{array}$$

**Case 6**

*P* = 1, *Q* = −(1 + *m* ^2^), *R* = *m* ^2^, *F*(*ξ*) = *dcξ*,

(20) $$\begin{array}{c} u_{6}=e^{i\theta }\left\{\pm 2\sqrt{-1-m^{2}-\alpha -3\alpha^{2}+3\beta }dc\xi \right\},\\ v_{6}=\alpha^{2}-\beta +1+m^{2}-2dc^{2}\xi . \end{array}$$

**Case 7**

*P* = 1 − *m* ^2^, *Q* = 2*m* ^2^ − 1, *R* = −*m* ^2^, *F*(*ξ*) = *ncξ*,

(21) $$\begin{array}{c} u_{7}=e^{i\theta }\left\{\pm 2\sqrt{\left(1-m^{2}\right)\left(2m^{2}-1-\alpha -3\alpha^{2}+3\beta \right)}nc\xi \right\},\\ v_{7}=\alpha^{2}-\beta -2m^{2}+1-2\left(1-m^{2}\right)nc^{2}\xi . \end{array}$$

**Case 8**

*P* = *m* ^2^ − 1, *Q* = 2 − *m* ^2^, *R* = −1, *F*(*ξ*) = *ndξ*,

(22) $$\begin{array}{c} u_{8}=e^{i\theta }\left\{\pm 2\sqrt{\left(m^{2}-1\right)\left(2-m^{2}-\alpha -3\alpha^{2}+3\beta \right)}nd\xi \right\},\\ v_{8}=\alpha^{2}-\beta -2+m^{2}-2\left(m^{2}-1\right)nd^{2}\xi . \end{array}$$

**Case 9**

*P* = 1 − *m* ^2^, *Q* = 2 − *m* ^2^, *R* = 1, *F*(*ξ*) = *scξ*,

(23) $$\begin{array}{c} u_{9}=e^{i\theta }\left\{\pm 2\sqrt{\left(1-m^{2}\right)\left(2-m^{2}-\alpha -3\alpha^{2}+3\beta \right)}sc\xi \right\},\\ v_{9}=\alpha^{2}-\beta -2+m^{2}-2\left(1-m^{2}\right)sc^{2}\xi . \end{array}$$

**Case 10**

*P* = −*m* ^2^(1 − *m* ^2^), *Q* = 2*m* ^2^ − 1, *R* = 1, *F*(*ξ*) = *sdξ*,

(24) $$\begin{array}{c} u_{10}=e^{i\theta }\left\{\pm 2m\sqrt{\left(m^{2}-1\right)\left(2m^{2}-1-\alpha -3\alpha^{2}+3\beta \right)}sd\xi \right\},\\ v_{10}=\alpha^{2}-\beta -2m^{2}+1+2m^{2}\left(1-m^{2}\right)sd^{2}\xi . \end{array}$$

**Case 11**

*P* = 1, *Q* = 2 − *m* ^2^, *R* = 1 − *m* ^2^, *F*(*ξ*) = *csξ*,

(25) $$\begin{array}{c} u_{11}=e^{i\theta }\left\{\pm 2\sqrt{2-m^{2}-\alpha -3\alpha^{2}+3\beta }cs\xi \right\},\\ v_{11}=\alpha^{2}-\beta -2+m^{2}-2cs^{2}\xi . \end{array}$$

**Case 12**

*P* = 1, *Q* = 2*m* ^2^ − 1, *R* = −*m* ^2^(1 − *m* ^2^), *F*(*ξ*) = *dsξ*,

(26) $$\begin{array}{c} u_{12}=e^{i\theta }\left\{\pm 2\sqrt{2m^{2}-1-\alpha -3\alpha^{2}+3\beta }ds\xi \right\},\\ v_{12}=\alpha^{2}-\beta -2m^{2}+1-2ds^{2}\xi . \end{array}$$

**Case 13**

*P* = 1/4, *Q* = (1 − 2*m* ^2^)/2, *R* = 1/4, *F*(*ξ*) = *nsξ* ± *csξ*,

(27) $$\begin{array}{c} u_{13}=e^{i\theta }\left\{\pm \sqrt{\frac{1-2m^{2}-2\alpha -6\alpha^{2}+6\beta }{2}}\left(ns\xi \pm cs\xi \right)\right\},\\ v_{13}=\frac{1}{2}\left\{2\alpha^{2}-2\beta -1+2m^{2}-{\left(ns\xi \pm cs\xi \right)}^{2}\right\}. \end{array}$$

**Case 14**

*P* = (1 − *m* ^2^)/4, *Q* = (1 + *m* ^2^)/2, *R* = (1 − *m* ^2^)/4, *F*(*ξ*) = *ncξ* ± *scξ*,

(28) u14=eiθ{±(1−m2)(1+m2−2α−6α2+6β)2   ×(ncξ±scξ)},v14=12{2α2−2β−1−m2−(1−m2)(ncξ±scξ)2}.

**Case 15**

*P* = 1/4, *Q* = (*m* ^2^ − 2)/2, *R* = *m* ^2^/4, *F*(*ξ*) = *nsξ* ± *dsξ*,

(29) $$\begin{array}{c} u_{15}=e^{i\theta }\left\{\pm \sqrt{\frac{m^{2}-2-2\alpha -6\alpha^{2}+6\beta }{2}}\left(ns\xi \pm ds\xi \right)\right\},\\ v_{15}=\frac{1}{2}\left\{2\alpha^{2}-2\beta -m^{2}+2-{\left(ns\xi \pm ds\xi \right)}^{2}\right\}. \end{array}$$

**Case 16**

*P* = *m* ^2^/4, *Q* = (*m* ^2^ − 2)/2, *R* = *m* ^2^/4, *F*(*ξ*) = *snξ* ± *i* *cnξ*,

(30) $$\begin{array}{c} u_{16}=e^{i\theta }\left\{\pm m\sqrt{\frac{m^{2}-2-2\alpha -6\alpha^{2}+6\beta }{2}}\left(sn\xi \pm icn\xi \right)\right\},\\ v_{16}=\frac{1}{2}\left\{2\alpha^{2}-2\beta -m^{2}+2-m^{2}{\left(sn\xi \pm icn\xi \right)}^{2}\right\}. \end{array}$$

**Case 17**

*P* = *m* ^2^/4, *Q* = (*m* ^2^ − 2)/2, *R* = *m* ^2^/4, $F(\xi )=\sqrt{1-m^{2}}sd\xi \pm cd\xi$,

(31) u17=eiθ{±mm2−2−2α−6α2+6β2×(1−m2sdξ±cdξ)},v17=12{2α2−2β−m2+2−m2(1−m2sdξ±cdξ)2}.

**Case 18**

*P* = 1/4, *Q* = (1 − *m* ^2^)/2, *R* = 1/4, $F(\xi )=mcd\xi \pm i\sqrt{1-m^{2}}nd\xi$,

(32) u18=eiθ{±1−m2−2α−6α2+6β2×(mcdξ±i1−m2ndξ)},v18=12{2α2−2β−1+m2−(mcdξ±i1−m2ndξ)2}.

**Case 19**

*P* = 1/4, *Q* = (1 − 2*m* ^2^)/2, *R* = 1/4, *F*(*ξ*) = *m* *snξ* ± *i* *dnξ*,

(33) u19=eiθ{±1−2m2−2α−6α2+6β2×(msnξ±idnξ)},v19=12{2α2−2β−1+2m2−(msnξ±idnξ)2}.

**Case 20**

*P* = 1/4, *Q* = (1 − *m* ^2^)/2, *R* = 1/4, $F(\xi )=\sqrt{1-m^{2}}sc\xi \pm idc\xi$,

(34) u20=eiθ{±1−m2−2α−6α2+6β2×(1−m2scξ±idcξ)},v20=12{2α2−2β−1+m2−(1−m2scξ±idcξ)2}.

**Case 21**

*P* = (*m* ^2^ − 1)/4, *Q* = (*m* ^2^ + 1)/2, *R* = (*m* ^2^ − 1)/4, *F*(*ξ*) = *m* *sdξ* ± *ndξ*,

(35) u21=eiθ{±(m2−1)(m2+1−2α−6α2+6β)2    ×(msdξ±ndξ)},v21=12{2α2−2β−m2−1−(m2−1)   ×(msdξ±ndξ)2}.

**Case 22**

*P* = *m* ^2^/4, *Q* = (*m* ^2^ − 2)/2, *R* = 1/4, *F*(*ξ*) = *snξ*/(1 ± *dnξ*),

(36) $$\begin{array}{c} u_{22}=e^{i\theta }\left\{\pm m\sqrt{\frac{m^{2}-2-2\alpha -6\alpha^{2}+6\beta }{2}}\frac{sn\xi }{1\pm dn\xi }\right\},\\ v_{22}=\frac{1}{2}\left\{2\alpha^{2}-2\beta -m^{2}+2-m^{2}{\left(\frac{sn\xi }{1\pm dn\xi }\right)}^{2}\right\}. \end{array}$$

**Case 23**

*P* = −1/4, *Q* = (*m* ^2^ + 1)/2, *R* = (1 − *m* ^2^)^2^/4, *F*(*ξ*) = *m* *cnξ* ± *dnξ*,

(37) $$\begin{array}{c} u_{23}=e^{i\theta }\left\{\pm \sqrt{\frac{-m^{2}-1+2\alpha +6\alpha^{2}-6\beta }{2}}\left(mcn\xi \pm dn\xi \right)\right\},\\ v_{23}=\frac{1}{2}\left\{2\alpha^{2}-2\beta -m^{2}-1+{\left(mcn\xi \pm dn\xi \right)}^{2}\right\}. \end{array}$$

**Case 24**

*P* = (1 − *m* ^2^)^2^/4, *Q* = (*m* ^2^ + 1)/2, *R* = 1/4, *F*(*ξ*) = *dsξ* ± *csξ*,

(38) u24=eiθ{±(1−m2)m2+1−2α−6α2+6β2×(dsξ±csξ)},v24=12{2α2−2β−m2−1−(1−m2)2(dsξ±csξ)2}.

**Case 25**

*P* = *m* ^4^(1 − *m* ^2^)/2(2 − *m* ^2^), *Q* = 2(1 − *m* ^2^)/(*m* ^2^ − 2), *R* = (1 − *m* ^2^)/2(2 − *m* ^2^), $F(\xi )=dc\xi \pm \sqrt{1-m^{2}}nc\xi$,

(39) u25 =eiθ{±2m22−m2×(m2−1)[2(1−m2)+(m2−2)(−α−3α2+3β)]×(dcξ±1−m2ncξ)},v25=1m2−2{(m2−2)(α2−β)+(1−m2)×[−2+m4(dcξ±1−m2ncξ)2]}.

**Case 26**

*P* > 0, *Q* < 0, *R* = *m* ^2^ *Q* ^2^/(1 + *m* ^2^)^2^ *P*, $F(\xi )=\sqrt{-m^{2}Q/(1+m^{2})P}sn\left(\sqrt{-Q/\left(1+m^{2}\right)}\xi \right)$,

(40) u26=eiθ{±2mQ(−Q+α+3α2−3β)1+m2×sn(−Q1+m2ξ)},v26=α2−β−Q+2m2Qm2+1sn2(−Q1+m2ξ).

**Case 27**

*P* < 0, *Q* > 0, *R* = (1 − *m* ^2^)*Q* ^2^/(*m* ^2^ − 2)^2^ *P*, $F(\xi )=\sqrt{-Q/(2-m^{2})P}dn\left(\sqrt{Q/\left(2-m^{2}\right)}\xi \right)$,

(41) u27=eiθ{±2Q(−Q+α+3α2−3β)2−m2×dn(Q2−m2ξ)},v27=α2−β−Q+2Q2−m2dn2(Q2−m2ξ).

**Case 28**

*P* < 0, *Q* > 0, *R* = *m* ^2^(*m* ^2^ − 1)*Q* ^2^/(2*m* ^2^ − 1)^2^ *P*, $F(\xi )=\sqrt{-m^{2}Q/(2m^{2}-1)P}cn\left(\sqrt{Q/\left(2m^{2}-1\right)}\xi \right)$,

(42) u28=eiθ{±2mQ(−Q+α+3α2−3β)2m2−1cn×(Q2m2−1ξ)},v28=α2−β−Q+2m2Q2m2−1cn2(Q2m2−1ξ).

**Case 29**

*P* = 1, *Q* = 2 − 4*m* ^2^, *R* = 1, *F*(*ξ*) = *snξ* *dnξ*/*cnξ*,

(43) $$\begin{array}{c} u_{29}=e^{i\theta }\left\{\pm 2\sqrt{2-4m^{2}-\alpha -3\alpha^{2}+3\beta }\frac{sn\xi dn\xi }{cn\xi }\right\},\\ v_{29}=\alpha^{2}-\beta -2+4m^{2}-\frac{2sn^{2}\xi dn^{2}\xi }{cn^{2}\xi }. \end{array}$$

**Case 30**

*P* = *m* ^2^, *Q* = 2, *R* = 1, *F*(*ξ*) = *snξ* *cnξ*/*dnξ*,

(44) $$\begin{array}{c} u_{30}=e^{i\theta }\left\{\pm 2m\sqrt{2-\alpha -3\alpha^{2}+3\beta }\frac{sn\xi cn\xi }{dn\xi }\right\},\\ v_{30}=\alpha^{2}-\beta -2-\frac{2m^{2}sn^{2}\xi cn^{2}\xi }{dn^{2}\xi }. \end{array}$$

**Case 31**

*P* = 1, *Q* = *m* ^2^ + 2, *R* = 1 − 2*m* ^2^ + *m* ^4^, *F*(*ξ*) = *dnξ* *cnξ*/*snξ*,

(45) $$\begin{array}{c} u_{31}=e^{i\theta }\left\{\pm 2\sqrt{m^{2}+2-\alpha -3\alpha^{2}+3\beta }\frac{dn\xi cn\xi }{sn\xi }\right\},\\ v_{31}=\alpha^{2}-\beta -m^{2}-2-\frac{2dn^{2}\xi cn^{2}\xi }{sn^{2}\xi }. \end{array}$$

**Case 32**

*P* = *A* ^2^(*m* − 1)^2^/4, *Q* = (*m* ^2^ + 1)/2 + 3*m*, *R* = (*m* − 1)^2^/4*A* ^2^, *F*(*ξ*) = *dnξ* *cnξ*/*A*(1 + *snξ*)(1 + *m* *snξ*),

(46) u32=eiθ{±(m−1)m2+6m+1−2α−6α2+6β2×dnξcnξ(1+snξ)(1+msnξ)},v32=12{2α2−2β−m2−6m−1−(m−1)2dn2ξcn2ξ(1+snξ)2(1+msnξ)2}.

**Case 33**

*P* = *A* ^2^(*m* + 1)^2^/4, *Q* = (*m* ^2^ + 1)/2 − 3*m*, *R* = (*m* + 1)^2^/4*A* ^2^, *F*(*ξ*) = *dnξ* *cnξ*/*A*(1 + *snξ*)(1 − *m* *snξ*),

(47) u33=eiθ{±(m+1)m2−6m+1−2α−6α2+6β2×dnξcnξ(1+snξ)(1−msnξ)},v33=12{2α2−2β−m2+6m−1−(m+1)2dn2ξcn2ξ(1+snξ)2(1−msnξ)2}.

**Case 34**

*P* = −4/*m*, *Q* = 6*m* − *m* ^2^ − 1, *R* = −2*m* ^3^ + *m* ^4^ + *m* ^2^, *F*(*ξ*) = *m* *cnξ* *dnξ*/(*msn* ^2^ *ξ* + 1),

(48) $$\begin{array}{c} u_{34}=e^{i\theta }\left\{\pm 4\sqrt{m^{2}-6m+1+\alpha +3\alpha^{2}-3\beta }\frac{\sqrt{m}cn\xi dn\xi }{msn^{2}\xi +1}\right\},\\ v_{34}=\alpha^{2}-\beta +m^{2}-6m+1+\frac{8mcn^{2}\xi dn^{2}\xi }{{\left(msn^{2}\xi +1\right)}^{2}}. \end{array}$$

**Case 35**

*P* = 4/*m*, *Q* = −6*m* − *m* ^2^ − 1, *R* = 2*m* ^3^ + *m* ^4^ + *m* ^2^, *F*(*ξ*) = *m* *cnξ* *dnξ*/(*msn* ^2^ *ξ* − 1),

(49) u35=eiθ{±4−m2−6m−1−α−3α2+3β×mcnξdnξmsn2ξ−1},v35=α2−β+m2+6m+1−8mcn2ξdn2ξ(msn2ξ−1)2.

**Case 36**

*P* = 1/4, *Q* = (1 − 2*m* ^2^)/2, *R* = 1/4, *F*(*ξ*) = *snξ*/(1 ± *cnξ*),

(50) $$\begin{array}{c} u_{36}=e^{i\theta }\left\{\pm \sqrt{\frac{1-2m^{2}-2\alpha -6\alpha^{2}+6\beta }{2}}\frac{sn\xi }{1\pm cn\xi }\right\},\\ v_{36}=\frac{1}{2}\left\{2\alpha^{2}-2\beta -1+2m^{2}-\frac{sn^{2}\xi }{{\left(1\pm cn\xi \right)}^{2}}\right\}. \end{array}$$

**Case 37**

*P* = (1 − *m* ^2^)/4, *Q* = (1 + *m* ^2^)/2, *R* = (1 − *m* ^2^)/4, *F*(*ξ*) = *cnξ*/(1 ± *snξ*),

(51) u37=eiθ{±(1−m2)(1+m2−2α−6α2+6β)2×cnξ1±snξ},v37=12{2α2−2β−1−m2−(1−m2)cn2ξ(1±snξ)2}.

**Case 38**

*P* = 4*m*_1_, *Q* = 2 + 6*m*_1_ − *m* ^2^, *R* = 2 + 2*m*_1_ − *m* ^2^, *F*(*ξ*) = *m* ^2^ *snξ* *cnξ*/(*m*_1_ − *dn* ^2^ *ξ*),

(52) u38=eiθ{±4m1(2+6m1−m2−α−3α2+3β)×m2snξcnξm1−dn2ξ},v38=α2−β−2−6m1+m2−8m1m4sn2ξcn2ξ(m1−dn2ξ)2.

**Case 39**

*P* = −4*m*_1_, *Q* = 2 − 6*m*_1_ − *m* ^2^, *R* = 2 − 2*m*_1_ − *m* ^2^, *F*(*ξ*) = −*m* ^2^ *snξ* *cnξ*/(*m*_1_ + *dn* ^2^ *ξ*),

(53) u39=eiθ{±4m1(−2+6m1+m2+α+3α2−3β)×m2snξcnξm1+dn2ξ},v39=α2−β−2+6m1+m2+8m1m4sn2ξcn2ξ(m1+dn2ξ)2.

**Case 40**

*P* = (2 − *m* ^2^ − 2*m*_1_)/4, *Q* = *m* ^2^/2 − 1 − 3*m*_1_, *R* = (2 − *m* ^2^ − 2*m*_1_)/4, *F*(*ξ*) = *m* ^2^ *snξ* *cnξ*/(*sn* ^2^ *ξ* + (1 + *m*_1_)*dnξ* − 1 − *m*_1_),

(54) u40 =eiθ{±(2−m2−2m1)(m2−2−6m1−2α−6α2+6β)2×m2snξcnξsn2ξ+(1+m1)dnξ−1−m1},v40=12{2α2−2β−m2+2+6m1−m4(2−m2−2m1)sn2ξcn2ξ[sn2ξ+(1+m1)dnξ−1−m1]2}.

**Case 41**

*P* = (2 − *m* ^2^ + 2*m*_1_)/4, *Q* = *m* ^2^/2 − 1 + 3*m*_1_, *R* = (2 − *m* ^2^ + 2*m*_1_)/4, *F*(*ξ*) = *m* ^2^ *snξ* *cnξ*/(*sn* ^2^ *ξ* + (−1 + *m*_1_)*dnξ* − 1 − *m*_1_),

(55) u41 =eiθ{±(2−m2+2m1)(m2−2+6m1−2α−6α2+6β)2×m2snξcnξsn2ξ+(−1+m1)dnξ−1−m1},v41=12{2α2−2β−m2+2−6m1−m4(2−m2+2m1)sn2ξcn2ξ[sn2ξ+(−1+m1)dnξ−1−m1]2}.

**Case 42**

*P* = (*C* ^2^ *m* ^4^ − (*B* ^2^ + *C* ^2^)*m* ^2^ + *B* ^2^)/4, *Q* = (*m* ^2^ + 1)/2, *R* = (*m* ^2^ − 1)/4(*C* ^2^ *m* ^2^ − *B* ^2^), $F(\xi )=\left(\sqrt{\left(B^{2}-C^{2}\right)/\left(B^{2}-C^{2}m^{2}\right)}+sn\xi \right)/\left(Bcn\xi +Cdn\xi \right)$,

(56) u42=eiθ{±[C2m4−(B2+C2)m2+B2](m2+1−2α−6α2+6β)2×(B2−C2)/(B2−C2m2)+snξBcnξ+Cdnξ},v42=12{2α2−2β−m2−1−[C2m4−(B2+C2)m2+B2]×((B2−C2)/(B2−C2m2)+snξBcnξ+Cdnξ)2}.

**Case 43**

*P* = (*B* ^2^ + *C* ^2^ *m* ^2^)/4, *Q* = 1/2 − *m* ^2^, *R* = 1/4(*B* ^2^ + *C* ^2^ *m* ^2^), $F(\xi )=\left(\sqrt{\left(C^{2}m^{2}+B^{2}-C^{2}\right)/\left(B^{2}+C^{2}m^{2}\right)}+cn\xi \right)/\left(Bsn\xi +Cdn\xi \right)$,

(57) u43=eiθ{±(B2+C2m2)(1−2m2−2α−6α2+6β)2×(C2m2+B2−C2)/(B2+C2m2)+cnξBsnξ+Cdnξ},v43=12{2α2−2β−1+2m2−(B2+C2m2)×((C2m2+B2−C2)/(B2+C2m2)+cnξBsnξ+Cdnξ)2}.

**Case 44**

*P* = (*B* ^2^ + *C* ^2^)/4, *Q* = *m* ^2^/2 − 1, *R* = *m* ^4^/4(*B* ^2^ + *C* ^2^), $F(\xi )=\left(\sqrt{\left(B^{2}+C^{2}-C^{2}m^{2}\right)/\left(B^{2}+C^{2}\right)}+dn\xi \right)/\left(Bsn\xi +Ccn\xi \right)$,

(58) u44=eiθ{±(B2+C2)(m2−2−2α−6α2+6β)2×(B2+C2−C2m2)/(B2+C2)+dnξBsnξ+Ccnξ},v44=12{2α2−2β−m2+2−(B2+C2)×((B2+C2−C2m2)/(B2+C2)+dnξBsnξ+Ccnξ)2}.

**Case 45**

*P* = −(*m* ^2^ + 2*m* + 1)*B* ^2^, *Q* = 2*m* ^2^ + 2, *R* = (2*m* − *m* ^2^ − 1)/*B* ^2^, *F*(*ξ*) = (*msn* ^2^ *ξ* − 1)/*B*(*msn* ^2^ *ξ* + 1),

(59) u45=eiθ{±2(m+1)−2m2−2+α+3α2−3β×msn2ξ−1msn2ξ+1},v45=α2−β−2m2−2+2(m+1)2×(msn2ξ−1msn2ξ+1)2.

**Case 46**

*P* = −(*m* ^2^ − 2*m* + 1)*B* ^2^, *Q* = 2*m* ^2^ + 2, *R* = −(2*m* + *m* ^2^ + 1)/*B* ^2^, *F*(*ξ*) = (*msn* ^2^ *ξ* + 1)/*B*(*msn* ^2^ *ξ* − 1),

(60) u46=eiθ{±2(m−1)−2m2−2+α+3α2−3β×msn2ξ+1msn2ξ−1},v46=α2−β−2m2−2+2(m−1)2×(msn2ξ+1msn2ξ−1)2.

We note that there is much duplication in the list of 46 solutions in terms of Jacobian-elliptic functions. Here are some examples; using the well-known identities relating Jacobian-elliptic functions (see 121.00, 129.01, 129.02, and 129.03 in [52], e.g.) reveals that *u*_1_, *u*_3_, and *u*_4_ are identical; *v*_1_, *v*_3_, and *v*_4_ are identical; *u*_2_, *u*_8_, and *u*_10_ are identical; *v*_2_, *v*_8_, and *v*_10_ are identical; *u*_5_, *u*_11_, and *u*_12_ are identical; *v*_5_, *v*_11_, and *v*_12_ are identical; *u*_6_, *u*_7_, and *u*_9_ are identical; *v*_6_, *v*_7_, and *v*_9_ are identical. Use of 162.01 in [52] reveals that *u*_27_ and *u*_28_ are equivalent and *v*_27_ and *v*_28_ are equivalent.

## 4. The New Weierstrass-Elliptic Function Solutions of the Schrödinger-KdV Equation

On using the solutions given in [43], mentioned in Appendix B, and from the formal solution (14) along with (10), we get then the following exact solutions.

**Case 47**

*g*_2_ = (4/3)(*Q* ^2^ − 3*PR*), *g*_3_ = (4*Q*/27)(−2*Q* ^2^ + 9*PR*), $F(\xi )=\sqrt{(1/P)\left[℘(\xi ;g_{2},g_{3})-(1/3)Q\right]}$,

(61) u47=eiθ{±2P(Q−α−3α2+3β)×1P[℘(ξ;g2,g3)−13Q]},v47=α2−β−Q−2[℘(ξ;g2,g3)−13Q].

**Case 48**

*g*_2_ = (4/3)(*Q* ^2^ − 3*PR*), *g*_3_ = (4*Q*/27)(−2*Q* ^2^ + 9*PR*), $F(\xi )=\sqrt{3R/\left(3℘(\xi ;g_{2},g_{3})-Q\right)}$,

(62) u48=eiθ{±2P(Q−α−3α2+3β)×3R3℘(ξ;g2,g3)−Q},v48=α2−β−Q−6PR3℘(ξ;g2,g3)−Q.

**Case 49**

*g*_2_ = −(5*QD* + 4*Q* ^2^ + 33*PQR*)/12, *g*_3_ = (21*Q* ^2^ *D* − 63*PR* *D* + 20*Q* ^3^ − 27*PQR*)/216, $F(\xi )=\sqrt{12R℘(\xi ;g_{2},g_{3})+2R(2Q+D)}/\left(12℘(\xi ;g_{2},g_{3})+D\right)$,

(63) u49=eiθ{±2P(Q−α−3α2+3β)    ×12R℘(ξ;g2,g3)+2R(2Q+D)12℘(ξ;g2,g3)+D},v49=α2−β−Q  −4PR[6℘(ξ;g2,g3)+2Q+D][12℘(ξ;g2,g3)+D]2.

**Case 50**

*g*_2_ = (1/12)*Q* ^2^ + *PR*, *g*_3_ = (1/216)*Q*(36*PR* − *Q* ^2^), $F(\xi )=\sqrt{R}\left[6℘\left(\xi ;g_{2},g_{3}\right)+Q\right]/3{℘}^{\prime }(\xi ;g_{2},g_{3})$,

(64) u50=eiθ{±2PR(Q−α−3α2+3β)×6℘(ξ;g2,g3)+Q3℘′(ξ;g2,g3)},v50=α2−β−Q−2PR[6℘(ξ;g2,g3)+Q]29[℘′(ξ;g2,g3)]2.

**Case 51**

*g*_2_ = (1/12)*Q* ^2^ + *PR*, *g*_3_ = (1/216)*Q*(36*PR* − *Q* ^2^), $F(\xi )=3{℘}^{\prime }\left(\xi ;g_{2},g_{3}\right)/\sqrt{P}\left[6℘\left(\xi ;g_{2},g_{3}\right)+Q\right]$,

(65) u51=eiθ{±2Q−α−3α2+3β3℘′(ξ;g2,g3)6℘(ξ;g2,g3)+Q},v51=α2−β−Q−18[℘′(ξ;g2,g3)]2[6℘(ξ;g2,g3)+Q]2.

**Case 52**

*R* = 5*Q* ^2^/36*P*, *g*_2_ = 2*Q* ^2^/9, *g*_3_ = *Q* ^3^/54, $F(\xi )=Q\sqrt{-15Q/2P}℘(\xi ;g_{2},g_{3})/\left(3℘(\xi ;g_{2},g_{3})+Q\right)$,

(66) u52=eiθ{±2P(Q−α−3α2+3β)×Q−15Q/2P℘(ξ;g2,g3)3℘(ξ;g2,g3)+Q},v52=α2−β−Q+15Q3℘2(ξ;g2,g3)[3℘(ξ;g2,g3)+Q]2.

It should be noted that any solution that can be expressed in terms of a Weierstrass-elliptic function can be also converted into a solution in terms of a Jacobian-elliptic function (for more details, see [53]). Consequently, Cases 47–52 are already covered in Cases 1–46. For example, using 1031.01 in [52] reveals that, with the *P*, *Q*, and *R* values for Case 1, *u*_1_ and *u*_48_ are identical and *v*_1_ and *v*_48_ are identical.

## 5. New Soliton-Like Solutions of the Schrödinger-KdV Equation

Some soliton-like solutions of (1) can be obtained in the limited case when the modulus *m* → 1 (see Appendix C), as follows:

(67) u1=eiθ{±2−2−α−3α2+3βtanhξ},v1=α2−β+2sech2ξ,u3=eiθ{±2−1+α+3α2−3βsechξ},v3=α2−β−1+2sech2ξ,u5=eiθ{±2−2−α−3α2+3βcoth⁡ξ},v5=α2−β−2csch2ξ,u11=eiθ{±21−α−3α2+3βcschξ},v11=α2−β−1−2csch2ξ,u13=eiθ{±−1−2α−6α2+6β2(coth⁡ξ±cschξ)},v13=12{2α2−2β+1−(coth⁡ξ±cschξ)2},u16=eiθ{±−1−2α−6α2+6β2(tanhξ±isechξ)},v16=12{2α2−2β+1−(tanhξ±isechξ)2},u22=eiθ{±−1−2α−6α2+6β2tanhξ1±sechξ},v22=12{2α2−2β+1−(tanhξ1±sechξ)2},u23=eiθ{±2−1+α+3α2−3βsechξ},v23=α2−β−1+2sech2ξ,u26=eiθ{±2Q(−Q+α+3α2−3β)2tanh(−Q2ξ)},v26=α2−β−Qsech2(−Q2ξ),u27=eiθ{±2Q(−Q+α+3α2−3β)sech(Qξ)},v27=α2−β−Q[1−2sech2(Qξ)],u30=eiθ{±22−α−3α2+3βtanhξ},v30=α2−β−2(2−sech2ξ),u31=eiθ{±23−α−3α2+3βsechξcschξ},v31=α2−β−3−2(sechξcschξ)2,u34=eiθ{±4−4+α+3α2−3βsech2ξ1+tanh2ξ},v34=α2−β−4+8sech4ξ(1+tanh2ξ)2,u40=eiθ{±(−1−2α−6α2+6β2)1+sechξ1−sechξ},v40=12{2α2−2β+1−1+sechξ1−sechξ},u41=eiθ{±(−1−2α−6α2+6β2)1−sechξ1+sechξ},v41=12{2α2−2β+1−1−sechξ1+sechξ},u43=eiθ{±−1−2α−6α2+6β2B+B2+C2sechξBtanhξ+Csechξ},v43=12{2α2−2β+1−(B+B2+C2sechξBtanhξ+Csechξ)2}.

Here, it should be noted that each exact solution given in (67) can be split into two solutions if one chooses the (+ve) and (−ve) signs, respectively, but they have not been calculated. Also, all the exact solutions given by (67) can be verified by substitution. The main feature for some of these exact solutions is the inclusion of the free parameters *Q*, *B*, and *C*.

## 6. New Trigonometric-Function Solutions of the Schrödinger-KdV Equation

Some trigonometric-function solutions of (1) can be obtained in the limited case when the modulus *m* → 0. For example,

(68) u5=eiθ{±2−1−α−3α2+3βcscξ},v5=α2−β+1−2csc2ξ,u6=eiθ{±2−1−α−3α2+3βsecξ},v6=α2−β+1−2sec2ξ,u9=eiθ{±22−α−3α2+3βtanξ},v9=α2−β−2sec2ξ,u11=eiθ{±22−α−3α2+3βcotξ},v11=α2−β−2csc2ξ,u13=eiθ{±1−2α−6α2+6β2(cscξ±cotξ)},v13=12{2α2−2β−1−(cscξ±cotξ)2},u14=eiθ{±1−2α−6α2+6β2(secξ±tanξ)},v14=12{2α2−2β−1−(secξ±tanξ)2},u24=eiθ{±1−2α−6α2+6β2(cscξ±cotξ)},v24=12{2α2−2β−1+(cscξ±cotξ)2},u32=eiθ{±1−2α−6α2+6β2(secξ−tanξ)},v32=α2−β−secξ(secξ−tanξ),u36=eiθ{±1−2α−6α2+6β2(cscξ±cotξ)},v36=12{2α2−2β−1−(cscξ±cotξ)2},u37=eiθ{±1−2α−6α2+6β2(secξ±tanξ)},v37=12{2α2−2β−1−(secξ±tanξ)2},u42=eiθ{±1−2α−6α2+6β2B2−C2+BsinξBcos⁡ξ+C},v42=12{2α2−2β−1−(B2−C2+BsinξBcos⁡ξ+C)2},u43=eiθ{±1−2α−6α2+6β2B2−C2+Bcos⁡ξBsinξ+C},v43=12{2α2−2β−1−(B2−C2+Bcos⁡ξBsinξ+C)2},u44=eiθ{±−1−α−3α2+3β2B2+C2Bsinξ+Ccos⁡ξ},v44=α2−β+1−2(B2+C2)(Bsinξ+Ccos⁡ξ)2.

Here, we note also that each trigonometric-function solution obtained in this section can split into two solutions if we choose the (+ve) and (−ve) signs, respectively. Besides, all these solutions can be verified by direct substitution. Also, the main feature for some of these exact solutions is the inclusion of the free parameters *Q*, *B*, and *C*.

## 7. Conclusion

In this paper, the *F*-expansion method has been applied to construct 52 types of exact solution of the the Schrödinger-KdV equation. The main advantage of this method over other methods is that it possesses all types of exact solution, including those of Jacobian-elliptic and Weierstrass-elliptic functions. Moreover, the soliton-like solutions and trigonometric-function solutions have been also obtained as the modulus *m* of Jacobi-elliptic function approaches to 1 and 0. It can be said that the results in this paper provide good supplements to the existing literature and are useful for describing certain nonlinear phenomena. This method can be applied to many other nonlinear evolution equations. Finally, it is worthwhile to mention that the proposed method is also a straightforward, short, promising, and powerful method for other nonlinear evolution equations in mathematical physics.

## Figures and Tables

**Table 1:** 

Case	*P*	*Q*	*R*	*F*(*ξ*)
1	*m* ^2^	−(1 + *m* ^2^)	1	*sn* *ξ*
2	*m* ^2^	−(1 + *m* ^2^)	1	$cd\xi =\frac{cn\xi }{dn\xi }$
3	−*m* ^2^	2*m* ^2^ − 1	1 − *m* ^2^	*cn* *ξ*
4	−1	2 − *m* ^2^	*m* ^2^ − 1	*dn* *ξ*
5	1	−(1 + *m* ^2^)	*m* ^2^	*ns* *ξ* = (*snξ*)^−1^
6	1	−(1 + *m* ^2^)	*m* ^2^	$dc\xi =\frac{dn\xi }{cn\xi }$
7	1 − *m* ^2^	2*m* ^2^ − 1	−*m* ^2^	*nc* *ξ* = (*cnξ*)^−1^
8	*m* ^2^ − 1	2 − *m* ^2^	−1	*nd* *ξ* = (*dnξ*)^−1^
9	1 − *m* ^2^	2 − *m* ^2^	1	$sc\xi =\frac{sn\xi }{cn\xi }$
10	−*m* ^2^(1 − *m* ^2^)	2*m* ^2^ − 1	1	$sd\xi =\frac{sn\xi }{dn\xi }$
11	1	2 − *m* ^2^	1 − *m* ^2^	$cs\xi =\frac{cn\xi }{sn\xi }$
12	1	2*m* ^2^ − 1	−*m* ^2^(1 − *m* ^2^)	$ds\xi =\frac{dn\xi }{sn\xi }$
13	$\frac{1}{4}$	$\frac{1-2m^{2}}{2}$	$\frac{1}{4}$	*ns* *ξ* ± *csξ*
14	$\frac{1-m^{2}}{4}$	$\frac{1+m^{2}}{2}$	$\frac{1-m^{2}}{4}$	*nc* *ξ* ± *scξ*
15	$\frac{1}{4}$	$\frac{m^{2}-2}{2}$	$\frac{m^{2}}{4}$	*ns* *ξ* ± *dsξ*
16	$\frac{m^{2}}{4}$	$\frac{m^{2}-2}{2}$	$\frac{m^{2}}{4}$	*sn* *ξ* ± *i* *cnξ*
17	$\frac{m^{2}}{4}$	$\frac{m^{2}-2}{2}$	$\frac{m^{2}}{4}$	$\sqrt{1-m^{2}}sd\xi \pm cd\xi$
18	$\frac{1}{4}$	$\frac{1-m^{2}}{2}$	$\frac{1}{4}$	$mcd\xi \pm i\sqrt{1-m^{2}}nd\xi$
19	$\frac{1}{4}$	$\frac{1-2m^{2}}{2}$	$\frac{1}{4}$	*m* *sn* *ξ* ± *i* *dnξ*
20	$\frac{1}{4}$	$\frac{1-m^{2}}{2}$	$\frac{1}{4}$	$\sqrt{1-m^{2}}sc\xi \pm dc\xi$
21	$\frac{m^{2}-1}{4}$	$\frac{m^{2}+1}{2}$	$\frac{m^{2}-1}{4}$	*ms* *dξ* ± *ndξ*
22	$\frac{m^{2}}{4}$	$\frac{m^{2}-2}{2}$	$\frac{1}{4}$	$\frac{sn\xi }{1\pm dn\xi }$
23	$-\frac{1}{4}$	$\frac{m^{2}+1}{2}$	$\frac{{\left(1-m^{2}\right)}^{2}}{4}$	*m* *cn* *ξ* ± *dnξ*
24	$\frac{{\left(1-m^{2}\right)}^{2}}{4}$	$\frac{m^{2}+1}{2}$	$\frac{1}{4}$	*ds* *ξ* ± *csξ*
25	$\frac{m^{4}(1-m^{2})}{2(2-m^{2})}$	$\frac{2(1-m^{2})}{m^{2}-2}$	$\frac{1-m^{2}}{2(2-m^{2})}$	$dc\xi \pm \sqrt{1-m^{2}}nc\xi$
26	*P* > 0	*Q* < 0	$\frac{m^{2}Q^{2}}{{\left(1+m^{2}\right)}^{2}P}$	$\sqrt{\frac{-m^{2}Q}{(1+m^{2})P}}sn\left(\sqrt{\frac{-Q}{1+m^{2}}}\xi \right)$
27	*P* < 0	*Q* > 0	$\frac{(1-m^{2})Q^{2}}{{\left(m^{2}-2\right)}^{2}P}$	$\sqrt{\frac{-Q}{(2-m^{2})P}}dn\left(\sqrt{\frac{Q}{2-m^{2}}}\xi \right)$
28	*P* < 0	*Q* > 0	$\frac{m^{2}(m^{2}-1)Q^{2}}{{\left(2m^{2}-1\right)}^{2}P}$	$\sqrt{-\frac{m^{2}Q}{(2m^{2}-1)P}}cn\left(\sqrt{\frac{Q}{2m^{2}-1}}\xi \right)$
29	1	2 − 4*m* ^2^	1	$\frac{sn\xi dn\xi }{cn\xi }$
30	*m* ^4^	2	1	$\frac{sn\xi cn\xi }{dn\xi }$
31	1	*m* ^2^ + 2	1 − 2*m* ^2^ + *m* ^4^	$\frac{dn\xi cn\xi }{sn\xi }$
32	$\frac{A^{2}{\left(m-1\right)}^{2}}{4}$	$\frac{m^{2}+1}{2}+3m$	$\frac{{\left(m-1\right)}^{2}}{4A^{2}}$	$\frac{dn\xi cn\xi }{A(1+sn\xi )(1+m\;\;sn\xi )}$
33	$\frac{A^{2}{\left(m+1\right)}^{2}}{4}$	$\frac{m^{2}+1}{2}-3m$	$\frac{{\left(m+1\right)}^{2}}{4A^{2}}$	$\frac{dn\xi cn\xi }{A(1+sn\xi )(1-m\;\;sn\xi )}$
34	$-\frac{4}{m}$	6*m* − *m* ^2^ − 1	−2*m* ^3^ + *m* ^4^ + *m* ^2^	$\frac{mcn\xi dn\xi }{msn^{2}\xi +1}$
35	$\frac{4}{m}$	−6*m* − *m* ^2^ − 1	2*m* ^3^ + *m* ^4^ + *m* ^2^	$\frac{mcn\xi dn\xi }{msn^{2}\xi -1}$
36	$\frac{1}{4}$	$\frac{1-2m^{2}}{2}$	$\frac{1}{4}$	$\frac{sn\xi }{1\pm cn\xi }$
37	$\frac{1-m^{2}}{4}$	$\frac{1+m^{2}}{2}$	$\frac{1-m^{2}}{4}$	$\frac{cn\xi }{1\pm sn\xi }$
38	4*m*_1_	2 + 6*m*_1_ − *m* ^2^	2 + 2*m*_1_ − *m* ^2^	$\frac{m^{2}sn\xi cn\xi }{m_{1}-dn^{2}\xi }$
39	−4*m*_1_	2 − 6*m*_1_ − *m* ^2^	2 − 2*m*_1_ − *m* ^2^	$-\frac{m^{2}sn\xi cn\xi }{m_{1}+dn^{2}\xi }$
40	$\frac{2-m^{2}-2m_{1}}{4}$	$\frac{m^{2}}{2}-1-3m_{1}$	$\frac{2-m^{2}-2m_{1}}{4}$	$\frac{m^{2}sn\xi cn\xi }{sn^{2}\xi +(1+m_{1})dn\xi -1-m_{1}}$
41	$\frac{2-m^{2}+2m_{1}}{4}$	$\frac{m^{2}}{2}-1+3m_{1}$	$\frac{2-m^{2}+2m_{1}}{4}$	$\frac{m^{2}sn\xi cn\xi }{sn^{2}\xi +(-1+m_{1})dn\xi -1+m_{1}}$
42	$\frac{C^{2}m^{4}-(B^{2}+C^{2})m^{2}+B^{2}}{4}$	$\frac{m^{2}+1}{2}$	$\frac{m^{2}-1}{4(C^{2}m^{2}-B^{2})}$	$\frac{\sqrt{\left((B^{2}-C^{2})/(B^{2}-C^{2}\;m^{2})\right)}+sn\xi }{Bcn\xi +Cdn\xi }$
43	$\frac{B^{2}+C^{2}m^{2}}{4}$	$\frac{1}{2}-m^{2}$	$\frac{1}{4(C^{2}m^{2}+B^{2})}$	$\frac{\sqrt{((C^{2}m^{2}+B^{2}-C^{2})/(B^{2}+C^{2}m^{2}))\;}+cn\xi }{Bsn\xi +Cdn\xi }$
44	$\frac{B^{2}+C^{2}}{4}$	$\frac{m^{2}}{2}-1$	$\frac{m^{4}}{4(C^{2}+B^{2})}$	$\frac{\sqrt{\left((B^{2}+C^{2}-C^{2}m^{2})/(B^{2}+C^{2})\right)}+dn\xi }{Bsn\xi +Ccn\xi }$
45	−(*m* ^2^ + 2*m* + 1)*B* ^2^	2*m* ^2^ + 2	$\frac{2m-m^{2}-1}{B^{2}}$	$\frac{msn^{2}\xi -1}{B(msn^{2}\xi +1)}$
46	−(*m* ^2^ − 2*m* + 1)*B* ^2^	2*m* ^2^ + 2	$-\frac{2m+m^{2}+1}{B^{2}}$	$\frac{msn^{2}\xi +1}{B(msn^{2}\xi -1)}$

**Table 2:** 

Case	*g*_2_	*g*_3_	*F*(*ξ*)
47	$\frac{4}{3}(Q^{2}-3PR)$	$\frac{4Q}{27}(-2Q^{2}+9PR)$	$\sqrt{\frac{1}{P}(℘(\xi ;g_{2},g_{3})-\frac{1}{3}Q)}$

48	$\frac{4}{3}(Q^{2}-3PR)$	$\frac{4Q}{27}(-2Q^{2}+9PR)$	$\sqrt{\frac{3R}{3℘(\xi ;g_{2},g_{3})-Q}}$

49	$-\frac{5QD+4Q^{2}+33PQR}{12}$	$\frac{21Q^{2}D-63PRD+20Q^{3}-27PQR}{216}$	$\frac{\sqrt{12R℘(\xi ;g_{2},g_{3})+2R(2Q+D)}}{12℘(\xi ;g_{2},g_{3})+D}$

50	$\frac{1}{12}Q^{2}+PR$	$\frac{1}{216}Q(36PR-Q^{2})$	$\frac{\sqrt{R}[6℘(\xi ;g_{2},g_{3})+Q]}{3{℘}^{\prime }(\xi ;g_{2},g_{3})}$

51	$\frac{1}{12}Q^{2}+PR$	$\frac{1}{216}Q(36PR-Q^{2})$	$\frac{3{℘}^{\prime }(\xi ;g_{2},g_{3})}{\sqrt{P}[6℘(\xi ;g_{2},g_{3})+Q]}$

52	$\frac{2Q^{2}}{9}$	$\frac{Q^{3}}{54}$	$\frac{Q\sqrt{-15Q/2P}℘(\xi ;g_{2},g_{3})}{3℘(\xi ;g_{2},g_{3})+Q}$, $R=\frac{5Q^{2}}{36P}$

## Appendices

### A. Relations between Values of (*P*, *Q*, *R*) and Corresponding *F*(*ξ*) in (7)

Relations between values of (*P*, *Q*, *R*) and corresponding *F*(*ξ*) in (7), where *A*, *B*, and *C* are arbitrary constants and $m_{1}=\sqrt{1-m^{2}}$. As shown in Table 1.

### B. The Weierstrass-Elliptic Function Solutions for (7)

The Weierstrass-elliptic function solutions for (7), where $D=(1/2)\left(-5Q\pm \sqrt{9Q^{2}-36PR}\right)$ and *℘*′(*ξ*; *g*_2_, *g*_3_) = *d℘*(*ξ*; *g*_2_, *g*_3_)/*dξ*. As shown in Table 2.

### C. Relations between Jacobian-Elliptic Functions and Hyperbolic Functions

The Jacobian-elliptic functions degenerate into hyperbolic functions when *m* → 1 as follows:

(C.1) snξ⟶tanhξ,  cnξ⟶sechξ,  dnξ⟶sechξ,scξ⟶sinh⁡ξ,  sdξ⟶sinh⁡ξ,  cdξ⟶1,nsξ⟶coth⁡ξ,  ncξ⟶cosh⁡ξ,  ndξ⟶cosh⁡ξ,csξ⟶cschξ,  dsξ⟶cschξ,  dcξ⟶1.

The Jacobian-elliptic functions degenerate into trigonometric functions when *m* → 0 as follows:

(C.2) snξ⟶sinξ,  cnξ⟶cos⁡ξ,  dnξ⟶1,scξ⟶tanξ,  sdξ⟶sinξ,  cdξ⟶cos⁡ξ,nsξ⟶cscξ,  ncξ⟶secξ,  ndξ⟶1,csξ⟶cotξ,  dsξ⟶cscξ,  dcξ⟶secξ.

### D. Some Trigonometric and Hyperbolic Identities

Consider the following:

(D.1) coth⁡θ−cschθ=tanhθ2,  cscθ−cotθ=tanθ2,coth⁡θ+cschθ=coth⁡θ2,  cscθ+cotθ=cotθ2,tanhθ+isechθ=tanh[12(θ+iπ2)],secθ+tanθ=tan[12(θ+π2)],tanhθ−isechθ=coth⁡[12(θ+iπ2)],secθ−tanθ=cot[12(θ+π2)].
